# Associations between DigComp 2.2-based digital competence and multidimensional health literacy among older adult learners in Shanghai Universities of the Third Age: a cross-sectional study

**DOI:** 10.3389/fpubh.2026.1883493

**Published:** 2026-08-31

**Authors:** Xindi Shen, Yingyi Luo, Chen Yang, Ouyang Xu, Yangli Wei, Qianqian Ma, Jiayu Cao

**Affiliations:** 1 Department of Arts and Science, Shanghai University of Medicine and Health Sciences, Shanghai, China; 2 College of Medical Technology, Shanghai University of Medicine and Health Sciences, Shanghai, China; 3 College of Continuing Education, Shanghai University of Medicine and Health Sciences, Shanghai, China; 4 Hospital Dean's Office, Cao He Jing Community Health Center, Shanghai, China; 5 Department of Nursing, Shanghai Jingan District Daning Road Sub-District Community Health Service Center, Shanghai, China; 6 Department of Nursing, Chuansha Community Health Service Center, Shanghai, China

**Keywords:** DigComp 2.2, digital competence, digital inclusion, Universities of the Third Age, healthy aging, HLQ, multidimensional health literacy, older adult learners

## Abstract

**Background:**

Digital competence may influence older adults' engagement with health information in increasingly digitalized societies. However, evidence on the association between DigComp 2.2-based digital competence and multidimensional health literacy remains limited. This study examined digital competence levels and its associations with health literacy domains among older adult learners in Shanghai Universities of the Third Age.

**Methods:**

A cross-sectional study was conducted from September to December 2024 in four public Universities of the Third Age in Shanghai, China. A total of 600 learners were recruited using convenience sampling, and 558 valid responses were analyzed. Digital competence was assessed using a 22-item DigComp 2.2-based scale covering five competence areas. Health literacy was measured using the 44-item Health Literacy Questionnaire (HLQ) with nine independent scales. Descriptive analysis, correlation analysis, and multiple linear regression were performed. Digital competence was modeled per 10-point increase.

**Results:**

The mean digital competence score was 63.4 (SD = 12.4), with communication and collaboration being the highest and digital content creation the lowest. HLQ scores varied across domains. All DigComp competence areas were positively correlated with HLQ scales (*r* = 0.285–0.538). Information and data literacy showed stronger correlations with health information-related HLQ domains. After adjustment, higher digital competence was significantly associated with appraisal of health information (*B* = 0.16, β = 0.284), navigating healthcare systems (*B* = 0.21, β = 0.312), finding good health information (*B = 0.24*, β = 0.338), and understanding health information (*B* = 0.19, β = 0.271; all *p* < 0.001).

**Conclusion:**

DigComp 2.2-based digital competence is positively associated with key domains of health literacy among older adult learners in Shanghai, particularly information appraisal, seeking, healthcare navigation, and understanding. Universities of the Third Age may serve as effective settings for integrated digital and health literacy education. Longitudinal and intervention studies are needed to confirm causality.

## 1 Introduction

Population aging and rapid digital transformation are reshaping the ways in which older adults access health information, communicate with health services, and participate in health-related decision-making (1–3). In this context, health literacy has become a key public health capacity in later life. It refers to the knowledge, motivation, and competencies needed to access, understand, appraise, and apply health information when making health-related decisions (4, 5). As health information and services are increasingly delivered through digital channels, digital competence has also become essential for older adults. It involves the confident, critical, responsible, and safe use of digital technologies for information seeking, communication, learning, problem solving, and social participation (6–8).

For older adults, the digital divide can exacerbate existing health disparities (9, 10). Individuals with limited digital competence may face barriers in accessing online health resources, using e-health portals, making medical appointments through digital platforms, or engaging with mobile health services (11–13). They may also encounter difficulties in evaluating the credibility of web-based health information, identifying misinformation, protecting personal data, or using digital health devices, which may limit their ability to obtain, appraise, and apply health information in everyday life (14–16).

Previous research has examined digital literacy, eHealth literacy, digital health literacy, and health literacy among older adults from different perspectives. Many studies have focused on digital access, frequency of internet use, internet-based health information seeking, or basic digital skills (17–19). However, fewer studies have examined digital competence as a multidimensional construct that includes not only access and operational skills, but also information evaluation, communication, content creation, safety, and problem solving. The European Digital Competence Framework for Citizens, DigComp 2.2, provides a multidimensional framework for understanding digital competence through five competence areas: information and data literacy, communication and collaboration, digital content creation, safety, and problem solving (20). These areas may be differently related to older adults' capacity to search for health information, communicate with health-related resources, protect personal information, and solve practical problems in digital health contexts (21).

The concept of health literacy has evolved considerably since Nutbeam's early conceptualization of it as a key outcome of health promotion and a tool for individual and community empowerment (22). It is now increasingly understood as a multidimensional construct rather than a single general ability. The Health Literacy Questionnaire (HLQ), originally developed and validated by Osborne et al., conceptualizes health literacy through nine independent domains, including support from health care providers, having sufficient information to manage health, actively managing health, social support for health, appraisal of health information, ability to engage with health care providers, navigating the health care system, ability to find good health information, and understanding health information well enough to know what to do (23). The Chinese version of the HLQ has been validated among older adults in China using confirmatory factor analysis (24, 25). As health services and information increasingly move online, Norman and Skinner (26) further proposed the concept of eHealth literacy to capture the specific skills required to seek, evaluate, and apply health information from digital sources. This multidimensional perspective is particularly relevant in digitalized healthcare systems, where older adults are expected not only to understand health information, but also to find, evaluate, communicate, and apply such information across both offline and online settings.

Universities of the Third Age are community-based learning institutions specifically designed for older adults, offering structured courses, social activities, and peer interaction to promote active aging and lifelong learning. In China, these institutions have emerged as important formal settings for later-life learning, providing structured opportunities for older adults to pursue knowledge, improve skills, and maintain social connections (27–29). Previous studies have shown that participation in Universities of the Third Age programs may support cognitive stimulation, psychosocial wellbeing, active aging, and lifelong learning (30–32). These institutions therefore attract older adults who are relatively active, socially engaged, and motivated to participate in educational activities (33). At the same time, learners from Universities of the Third Age may still face barriers in using digital technologies for health-related purposes, such as making online medical appointments, identifying reliable health information, using mobile health services, or protecting personal information. However, it is important to note that learners from Universities of the Third Age represent a selected subgroup of older adults who are relatively active, educated, and motivated. Therefore, the findings of this study are not directly generalizable to the entire older adult population in China. Universities of the Third Age may therefore represent an important and educationally reachable public health learning setting for supporting digital inclusion and health literacy among older adults.

Despite the growing literature, several gaps remain. First, existing studies have often focused on digital access, internet use, eHealth literacy, or digital health literacy among older adults, whereas less attention has been paid to DigComp-based multidimensional digital competence in this population (34–36). Second, previous research has mainly examined community-dwelling older adults, rural older adults, patients, or older adults living alone, while older adult learners enrolled in Universities of the Third Age remain underexplored (37–39). Boulton-Lewis (40) emphasized that understanding older learners' educational needs is critical for designing effective programmes, yet digital competence in this specific group has received little attention. Third, limited evidence exists on how specific domains of digital competence are associated with specific domains of health literacy, particularly when health literacy is conceptualized as a multidimensional construct using instruments such as the HLQ (23, 24). This gap is important because digital competence may not be equally related to all aspects of health literacy. Based on the conceptual alignment between DigComp 2.2 and the HLQ, information and data literacy may be closely related to finding and appraising health information; communication and collaboration may support interaction with health care providers and social support for health; safety may be relevant to judging the reliability of online information and protecting personal data; and problem solving may support navigation of digital health services and the healthcare system.

Guided by this conceptual framework, the present study aimed to investigate DigComp 2.2-based digital competence and multidimensional health literacy among older adult learners in Shanghai Universities of the Third Age, and to examine the associations between digital competence and specific HLQ domains. By linking the five areas of digital competence with the nine domains of health literacy, this study seeks to provide a more nuanced understanding of how digital capabilities are related to health literacy in later-life learning settings. From a public health perspective, the findings may inform the development and future evaluation of integrated digital skills and health education programs aimed at reducing digital exclusion, supporting health literacy, and contributing to healthy aging among older adults in rapidly digitalizing societies (41, 42).

## 2 Materials and methods

### 2.1 Study design and participants

A cross-sectional study was conducted in four large public community Universities of the Third Age in Shanghai, China, located in Xuhui District, Jing'an District, Pudong New Area, and Yangpu District. These sites were selected to cover different urban districts and to enhance the geographic diversity of the sample. Data collection was conducted from September to December 2024. Participants were recruited using convenience sampling.

The inclusion criteria were as follows: (1) aged 55 years or older, consistent with the enrollment profile of Universities of the Third Age in China; (2) enrolled as a student in a University of the Third Age for at least one semester; (3) having adequate cognitive ability to provide informed consent and complete the questionnaire; and (4) willingness to participate voluntarily. Individuals with severe visual, hearing, or cognitive impairments that prevented them from completing the questionnaire were excluded.

This study was reported in accordance with the Strengthening the Reporting of Observational Studies in Epidemiology (STROBE) guidelines for cross-sectional studies. Sample size was calculated a priori using G^*^Power 3.1 (43) for multiple linear regression. Based on an anticipated medium effect size (*f*^2^ = 0.15), α = 0.05, power = 0.95, and up to 10 candidate independent variables or covariates planned for inclusion in the regression models, a minimum sample of 381 was required. The final sample of 558 exceeded this requirement.

### 2.2 Measures

A socio-demographic and health-related questionnaire was used to collect information on age, gender, educational attainment, former occupation, monthly income, self-rated health, and number of chronic conditions. Self-rated health was assessed on a five-point Likert scale ranging from 1 = very poor to 5 = very good. The number of chronic conditions was self-reported and categorized as 0, 1, or ≥2 chronic conditions.

Digital competence was measured using a 22-item scale developed based on the European Digital Competence Framework for Citizens, DigComp 2.2 (20). DigComp 2.2 conceptualizes digital competence as a multidimensional construct consisting of five competence areas and 21 specific competences. The five competence areas are information and data literacy, communication and collaboration, digital content creation, safety, and problem solving.

The items were adapted to reflect older adult learners' everyday digital technology use and health-related digital contexts, such as searching for online information, communicating through digital tools, protecting personal information, and solving common problems when using digital services. The wording of the items was reviewed by the research team to ensure relevance and readability for older adult learners. Each item was rated on a five-point Likert scale ranging from 1 = strongly disagree to 5 = strongly agree.

The total score was calculated as the sum of all 22 items, ranging from 22 to 110, with higher scores indicating higher overall digital competence. Competence area scores were calculated as the mean score of items within each area, with higher mean scores indicating higher competence in the corresponding area. Prior to the main survey, the questionnaire was piloted with 50 older adult learners to assess clarity, relevance, and readability, and items were revised based on their feedback. In the present study, the Cronbach's α coefficient for the total scale was 0.910, and Cronbach's α coefficients for the five competence areas ranged from 0.833 to 0.889.

Health literacy was measured using the Chinese version of the Health Literacy Questionnaire (HLQ). The original HLQ was developed and validated by Osborne et al. as a multidimensional instrument for assessing health literacy across different domains (23). The Chinese version of the HLQ has been validated among older adults in China by Huang et al. using confirmatory factor analysis (24). A licence to use the Chinese version of the HLQ was obtained from the original HLQ authors (Osborne et al.) under licence agreement number L26061. The questionnaire was administered according to the licence conditions, and no modifications were made to the original HLQ items.

The HLQ consists of 44 items across nine independent scales: (1) feeling understood and supported by health care providers (4 items); (2) having sufficient information to manage health (4 items); (3) actively managing health (5 items); (4) social support for health (5 items); (5) appraisal of health information (5 items); (6) ability to actively engage with health care providers (5 items); (7) navigating the health care system (6 items); (8) ability to find good health information (5 items); and (9) understanding health information well enough to know what to do (5 items).

The first five scales use a four-point Likert-type response format ranging from 1 = strongly disagree to 4 = strongly agree, whereas scales 6 to 9 use a five-point Likert-type response format ranging from 1 = cannot do or always difficult to 5 = always easy. Following the standard HLQ scoring approach, each of the nine scale scores was calculated as the mean score of the items within that scale. Scales 1 to 5 therefore have a possible score range of 1 to 4, while scales 6 to 9 have a possible score range of 1 to 5. Higher scores indicate higher health literacy in the corresponding domain.

Because the HLQ is designed to provide nine independent scale scores rather than a single total score, the 44 items were not summed into an overall HLQ score. No standardized 0–100 score or cut-off for adequate health literacy was used. In the present study, Cronbach's α coefficients for the nine HLQ scales ranged from 0.780 to 0.881.

### 2.3 Data collection and ethical considerations

The study was conducted in accordance with the Declaration of Helsinki (44). Ethical approval was obtained from the Institutional Review Board of Shanghai University of Medicine and Health Sciences. All participants provided written informed consent before participation.

Trained research assistants distributed paper questionnaires on site. Before completing the questionnaire, participants were informed of the study purpose, voluntary nature of participation, confidentiality of responses, and their right to withdraw at any time. Research assistants provided standardized explanations and necessary assistance to participants who had difficulty reading or understanding the questionnaire, without influencing their responses.

Returned questionnaires were checked for completeness and response quality. Questionnaires with substantial missing data or invalid response patterns were excluded before analysis.

### 2.4 Statistical analysis

Data were analyzed using IBM SPSS Statistics version 26.0. Descriptive statistics were used to summarize participant characteristics, DigComp 2.2-based digital competence scores, and the nine HLQ scale scores. Continuous variables were presented as means and standard deviations, and categorical variables were presented as frequencies and percentages. Internal consistency was assessed using Cronbach's α coefficients.

For digital competence, the total score was calculated as the sum of all 22 items, and the five competence area scores were calculated as mean scores of items within each area. For the HLQ, each scale score was calculated separately as the mean score of the items within that scale. Because the HLQ is designed to provide nine independent scale scores rather than a single total score, the nine HLQ scale scores were analyzed separately.

Pearson correlation analyses were conducted to examine associations between the mean scores of the five DigComp competence areas and the nine HLQ scale scores. The results were visualized using a heatmap, with the color intensity representing the magnitude of the correlation coefficients. Individual correlation values were annotated within the heatmap cells. Because the dimension-level correlation analyses were exploratory, p-values were interpreted cautiously, with emphasis on the magnitude, direction, and consistency of associations.

Multiple linear regression analyses were conducted to examine the associations between digital competence and selected HLQ outcomes after adjustment for socio-demographic and health-related covariates. Based on conceptual relevance to digital health contexts (45, 46), four information-related HLQ scales were selected as dependent variables in the regression analyses: appraisal of health information, navigating the health care system, ability to find good health information, and understanding health information well enough to know what to do. These domains were chosen because they directly reflect older adults' capacity to search, evaluate, understand, and apply health information, which are theoretically most likely to be influenced by digital competence. The main independent variable was overall digital competence. For regression analyses, the overall digital competence score was divided by 10 to facilitate interpretation of unstandardized coefficients. Covariates included age, gender, educational attainment, monthly income, self-rated health, and number of chronic conditions. Additional exploratory regression models were conducted using the five DigComp competence area scores as independent variables to examine which areas of digital competence were most closely associated with selected HLQ domains. Variance inflation factors (VIFs) were examined to assess multicollinearity. A two-tailed p-value < 0.05 was considered statistically significant.

## 3 Results

### 3.1 Participant characteristics

A total of 600 questionnaires were distributed, and 558 valid responses were included in the analysis. As shown in Table 1, 238 participants were male (42.7%) and 320 were female (57.3%). Participants aged 55–59 years accounted for the largest proportion of the sample (36.4%), followed by those aged 60–69 years (33.9%), 70–79 years (21.7%), and 80 years or older (8.1%). More than two thirds of participants had a high school education or above, and 291 participants (52.1%) reported good or very good self-rated health. The mean overall digital competence score was 63.4 (SD = 12.4). Digital competence scores differed significantly by age group, educational level, monthly income, self-rated health, and number of chronic conditions. Participants aged 55–59 years had the highest digital competence score, whereas those aged 80 years or older had the lowest score. Higher educational attainment, higher monthly income, and better self-rated health were associated with higher digital competence scores. Participants with no chronic conditions also had higher digital competence scores than those with one or more chronic conditions. No statistically significant gender difference was observed in digital competence scores.

**Table 1:** Sociodemographic and health-related characteristics of older adult learners and overall digital competence scores (*N* = 558).

Characteristic	Total, *n* (%)	Digital competence, Mean (SD)	*p*-value
Gender			
Male	238 (42.7)	64.8 (11.9)	0.064
Female	320 (57.3)	62.4 (12.6)	
Age group, years			< 0.001
55–59	203 (36.4)	67.4 (11.4)	
60–69	189 (33.9)	66.3 (11.5)	
70–79	121 (21.7)	60.1 (12.0)	
≥80	45 (8.1)	42.1 (10.7)	
Education level			< 0.001
Primary school or below	78 (14.0)	55.1 (10.2)	
Middle school	98 (17.6)	60.8 (11.1)	
High school	195 (34.9)	64.2 (11.5)	
College or above	187 (33.5)	68.0 (12.3)	
Monthly income, CNY			< 0.001
< 3,000	67 (12.0)	57.9 (11.8)	
3,000–5,999	277 (49.6)	62.5 (11.9)	
≥6,000	214 (38.4)	66.4 (12.0)	
Self-rated health			< 0.001
Poor/very poor	89 (16.0)	57.3 (13.2)	
Fair	178 (31.9)	61.6 (11.5)	
Good/very good	291 (52.1)	66.9 (11.4)	
Number of chronic conditions			0.021
0	187 (33.5)	65.5 (12.1)	
1	223 (40.0)	63.2 (12.0)	
≥2	148 (26.5)	61.0 (12.8)	
Overall	558 (100)	63.4 (12.4)	

CNY, Chinese Yuan; SD, standard deviation. *p*-values were derived from independent samples *t*-test for dichotomous variables and one-way ANOVA for categorical variables with more than two groups. All *p*-values are unadjusted for multiple comparisons.

### 3.2 Digital competence and HLQ scale scores

As shown in Table 2, the mean overall digital competence score was 63.4 (SD = 12.4). Among the five DigComp 2.2 competence areas, communication and collaboration had the highest mean score, whereas digital content creation had the lowest mean score. The DigComp 2.2-based scale showed good internal consistency, with a Cronbach's α of 0.910 for the total scale and Cronbach's α coefficients ranging from 0.833 to 0.889 across the five competence areas.

**Table 2:** Descriptive statistics and internal consistency of DigComp 2.2 competence areas and HLQ scales.

Measure/domain	No. of items	Score range	Mean (SD)	Cronbach's α
Digital competence total score	22	22–110	63.4 (12.4)	0.910
Information and data literacy	5	1–5	2.9 (0.8)	0.842
Communication and collaboration	5	1–5	3.2 (0.8)	0.861
Digital content creation	4	1–5	2.5 (0.9)	0.889
Safety	4	1–5	3.0 (0.7)	0.833
Problem solving	4	1–5	2.8 (0.8)	0.872
HLQ1 Feeling understood and supported by health care providers	4	1–4	3.1 (0.7)	0.805
HLQ2 Having sufficient information to manage health	4	1–4	2.9 (0.7)	0.813
HLQ3 Actively managing health	5	1–4	2.7 (0.7)	0.780
HLQ4 Social support for health	5	1–4	3.1 (0.7)	0.823
HLQ5 Appraisal of health information	5	1–4	2.5 (0.8)	0.881
HLQ6 Ability to actively engage with health care providers	5	1–5	3.5 (0.8)	0.828
HLQ7 Navigating the health care system	6	1–5	3.3 (0.8)	0.880
HLQ8 Ability to find good health information	5	1–5	2.9 (0.8)	0.808
HLQ9 Understanding health information well enough to know what to do	5	1–5	3.1 (0.8)	0.799

The overall digital competence score was calculated as the sum of all 22 items. DigComp competence area scores were calculated as mean scores of items within each area. HLQ scale scores were calculated as mean scores of items within each scale. HLQ1–HLQ5 have possible score ranges of 1–4, whereas HLQ6–HLQ9 have possible score ranges of 1–5. Higher scores indicate higher digital competence or health literacy in the corresponding domain.

The HLQ scale scores varied across domains. Among HLQ1–HLQ5, which use a four-point response format, the highest mean scores were observed for feeling understood and supported by health care providers and social support for health, whereas the lowest score was observed for appraisal of health information. Among HLQ6–HLQ9, which use a five-point response format, the highest mean score was observed for ability to actively engage with health care providers, whereas the lowest was observed for ability to find good health information. Cronbach's α coefficients for the nine HLQ scales ranged from 0.780 to 0.881, indicating acceptable to good internal consistency.

### 3.3 Correlations between DigComp competence areas and HLQ scales

**Figure 1:**
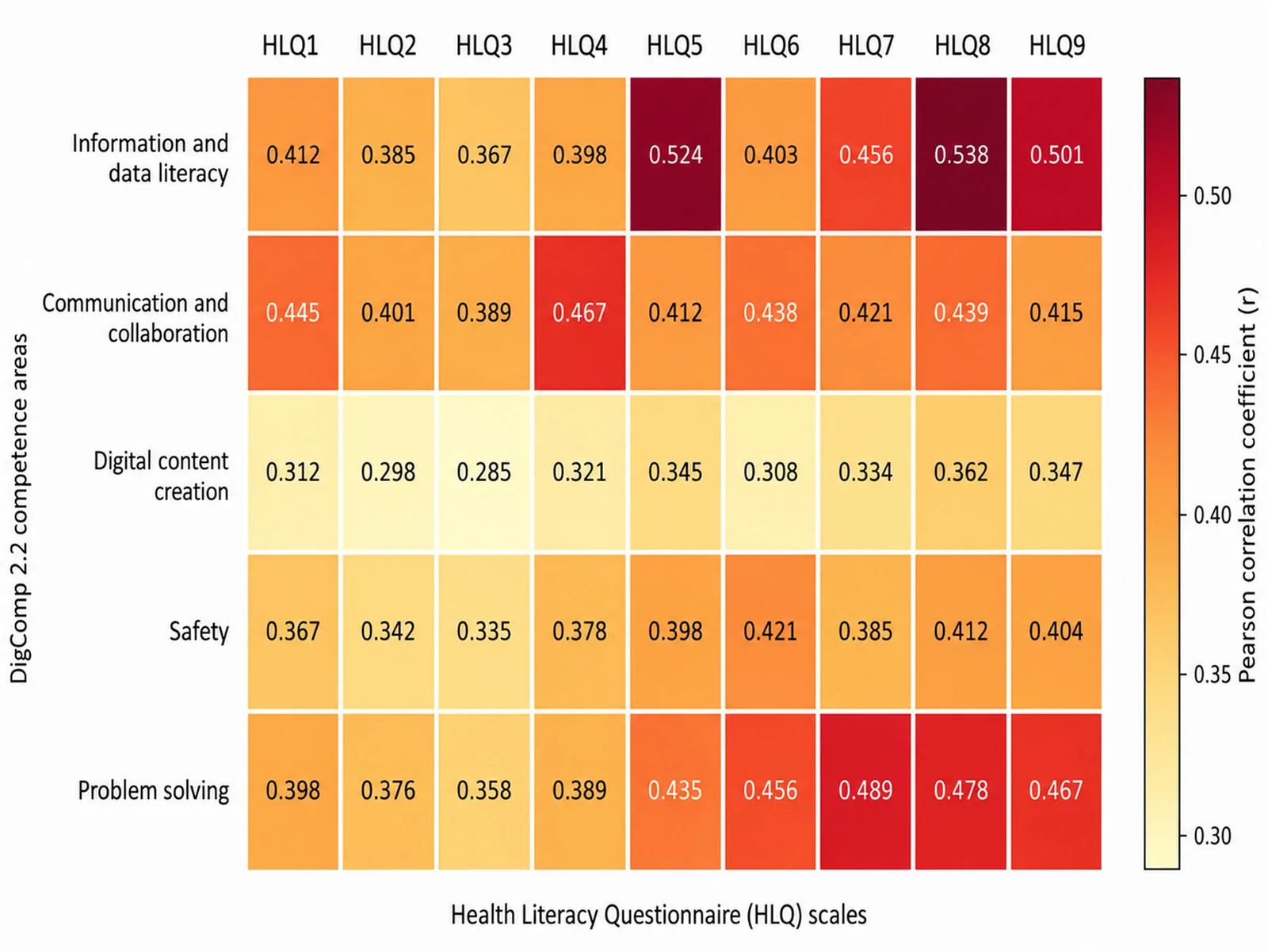
Heatmap of correlations between DigComp 2.2 competence areas and HLQ scales. Cells show Pearson correlation coefficients between the five DigComp 2.2 competence areas and the nine HLQ scales. Darker colors indicate stronger positive correlations. Values displayed in the cells are correlation coefficients. HLQ1 = feeling understood and supported by health care providers; HLQ2 = having sufficient information to manage health; HLQ3 = actively managing health; HLQ4 = social support for health; HLQ5 = appraisal of health information; HLQ6 = ability to actively engage with health care providers; HLQ7 = navigating the health care system; HLQ8 = ability to find good health information; HLQ9 = understanding health information well enough to know what to do. All correlations were statistically significant at *p* < 0.01.

Figure 1 shows the correlations between the five DigComp 2.2 competence areas and the nine HLQ scales. All competence areas were positively associated with health literacy domains, with correlation coefficients ranging from 0.285 to 0.538, indicating weak to moderate relationships across domains.

Information and data literacy exhibited relatively stronger correlations with information-related HLQ scales, including appraisal of health information (r = 0.524, *p* < 0.01), ability to find good health information (r = 0.538, *p* < 0.01), and understanding health information well enough to know what to do (r = 0.501, *p* < 0.01). Problem solving was also positively correlated with navigating the health care system (r = 0.489, *p* < 0.01), ability to find good health information (r = 0.478, *p* < 0.01), and understanding health information well enough to know what to do (r = 0.467, *p* < 0.01).

Digital content creation showed comparatively weaker, but still significant, associations with all HLQ scales. The heatmap in Figure 1 provides a visual representation of the magnitude and direction of these correlations, with higher correlation coefficients indicated by darker shades.

### 3.4 Associations between overall digital competence and selected HLQ domains

As shown in Table 3, overall digital competence was positively associated with all four selected HLQ domains after adjustment for socio-demographic and health-related covariates. Each 10-point higher overall digital competence score was associated with a 0.16-point higher score in appraisal of health information, a 0.21-point higher score in navigating the health care system, a 0.24-point higher score in ability to find good health information, and a 0.19-point higher score in understanding health information well enough to know what to do. The strongest adjusted association was observed for the ability to find good health information.

**Table 3:** Adjusted associations between overall digital competence and selected HLQ domains among older adult learners.

Dependent variable	B (95% CI) per 10-point increase	β	*p* value	Adjusted R^2^
HLQ5 Appraisal of health information	0.16 (0.11, 0.21)	0.284	< 0.001	0.198
HLQ7 Navigating the health care system	0.21 (0.15, 0.27)	0.312	< 0.001	0.246
HLQ8 Ability to find good health information	0.24 (0.18, 0.30)	0.338	< 0.001	0.287
HLQ9 Understanding health information well enough to know what to do	0.19 (0.13, 0.25)	0.271	< 0.001	0.215

Each row represents a separate multiple linear regression model. The main independent variable was overall digital competence, expressed per 10-point increase. Models were adjusted for age, gender, educational attainment, monthly income, self-rated health, and number of chronic conditions. Adjusted R^2^ refers to the explanatory power of the full adjusted model. B, unstandardized coefficient; CI, confidence interval; β, standardized coefficient; HLQ, Health Literacy Questionnaire.

## 4 Discussion

This study examined the associations between DigComp 2.2-based digital competence and multidimensional health literacy among older adult learners in Shanghai Universities of the Third Age. The findings showed that digital competence was positively associated with several HLQ domains, particularly those related to appraising health information, finding good health information, navigating the health care system, and understanding health information well enough to know what to do. These domains are closely linked to older adults' everyday health information practices in increasingly digitalized healthcare environments. The results suggest that digital competence may be an important factor to consider in public health education for older adult learners, especially when health information and health services are increasingly delivered through digital channels. However, because of the cross-sectional design, these associations should not be interpreted as evidence of a causal relationship.

### 4.1 Main findings

The present study provides a more nuanced understanding of the relationship between digital competence and health literacy by examining both constructs as multidimensional. Rather than treating health literacy as a single total score, this study used the nine independent domains of the HLQ. The results showed that HLQ scores varied across domains, indicating that older adult learners may have different strengths and needs in different aspects of health literacy. In particular, relatively lower scores were observed in appraisal of health information and ability to find good health information. These findings suggest that even among socially active older learners enrolled in Universities of the Third Age, challenges remain in identifying, evaluating, and using reliable health information.

The dimension-level correlation analysis further showed that all five DigComp 2.2 competence areas were positively correlated with the nine HLQ scales, but the magnitude of associations varied across domains. Information and data literacy showed relatively stronger correlations with appraisal of health information, ability to find good health information, and understanding health information well enough to know what to do. Problem solving was also closely associated with navigating the health care system and understanding health information. In contrast, digital content creation showed comparatively weaker correlations with HLQ domains. This pattern is theoretically plausible because older adults' routine digital health tasks are more likely to involve searching for information, judging its reliability, using digital health services, and solving practical problems, rather than creating digital content.

The adjusted regression analyses also supported the relevance of digital competence to selected HLQ domains. After adjustment for socio-demographic and health-related covariates, each 10-point higher overall digital competence score was positively associated with higher scores in appraisal of health information, navigating the health care system, ability to find good health information, and understanding health information well enough to know what to do. The strongest adjusted association was observed for the ability to find good health information. These findings indicate that digital competence is particularly relevant to health literacy tasks that require older adults to locate, evaluate, interpret, and apply health information in daily life and healthcare settings.

### 4.2 Public health significance: digital competence, health literacy, and health equity

The findings have important public health implications. In digitalized societies, health literacy is no longer limited to reading health materials or understanding medical instructions. Older adults are increasingly expected to use smartphones, online appointment systems, electronic health information, mobile payment systems, and web-based health services, which may create new forms of digital health inequality for those with limited digital competence (45, 47, 48). As a result, insufficient digital competence may contribute to unequal access to health information and services, thereby reinforcing existing health disparities.

From this perspective, digital competence should be understood not only as a technical skill, but also as a public health-related capability that may influence access to health information, service navigation, and participation in health-related decision-making (46, 49). Older adults with stronger information and data literacy may be better able to locate reliable health information and distinguish credible sources from misinformation. Those with stronger problem-solving competence may be better able to overcome practical barriers when using online medical services, navigating hospital platforms, or seeking health-related support. Therefore, improving digital competence may be a promising direction for health literacy promotion, although longitudinal and intervention studies are needed to verify whether changes in digital competence lead to subsequent changes in health literacy.

This study also suggests that health literacy interventions for older adults should pay particular attention to information appraisal, information seeking, and healthcare navigation. These domains are highly relevant to digital health equity. Older adults who cannot effectively search for reliable information, evaluate online health claims, or navigate digital health services may be disadvantaged in accessing timely and appropriate healthcare. Public health programs aimed at reducing the digital divide should therefore move beyond basic device training and include health information evaluation, privacy protection, digital service navigation, and practical problem solving.

A critical equity gap: older adults with cognitive impairment. It is important to acknowledge that our study did not include older adults with cognitive impairment, such as those with mild cognitive impairment or dementia. This group faces unique barriers that cannot be addressed through conventional digital skills training. If digital literacy becomes a prerequisite for accessing essential health services, including online appointment systems, electronic health records, and telemedicine, a large segment of the cognitively impaired older population may be systematically excluded from equitable healthcare regardless of educational efforts. In such cases, achieving digital health equity may indeed be impossible through standard digital inclusion strategies. Therefore, alongside improving digital competence among cognitively intact older adults, policymakers and healthcare systems must preserve and strengthen non-digital access routes, such as telephone appointments, in-person services, caregiver-supported navigation, and assistive technologies, to ensure that cognitively impaired older adults are not left behind. Future research should specifically investigate the digital health needs, barriers, and potential accommodations for this underserved population.

### 4.3 Universities of the Third Age as feasible public health learning settings

A key contribution of this study is its focus on learners from Universities of the Third Age. Universities of the Third Age are not merely institutions for leisure learning; they may also contribute to active aging, social participation, cognitive stimulation, and community-based health promotion (33, 50). Compared with older adults who are socially isolated or difficult to reach, learners from Universities of the Third Age participate in structured learning activities and have regular opportunities for peer interaction, teacher support, and curriculum-based engagement. This makes Universities of the Third Age a potentially feasible setting for delivering integrated digital skills and health education programs.

The present findings support the value of using Universities of the Third Age as intervention platforms. First, the results indicate that older learners still have specific health literacy needs, particularly in appraising and finding health information. Second, digital competence was positively associated with these health literacy domains, suggesting that digital skills may be an important educational target. Third, the setting of Universities of the Third Age provides a practical environment in which digital health literacy courses, workshops, peer-assisted learning, and scenario-based training can be implemented. For example, courses may include modules on identifying reliable online health information, avoiding health misinformation and digital fraud, using online appointment systems, protecting personal health data, communicating with healthcare providers through digital channels, and navigating hospital or community health platforms.

These implications are directly relevant to public health practice. Community-based health promotion often faces the challenge of reaching older adults in sustained and organized ways. Universities of the Third Age may help bridge this gap by providing a stable learning community. Integrating digital competence training into Universities of the Third Age curricula could support digital inclusion, strengthen older adults' ability to use health information, and contribute to healthy aging. Such programs may also be linked with community health centers, family doctor services, public health campaigns, and local aging services, thereby extending the impact of education in Universities of the Third Age into broader community health promotion.

### 4.4 Comparison with prior studies and possible explanations

The findings are consistent with previous studies showing that digital skills, eHealth literacy, and online health information use are related to older adults' ability to access and use health information (13, 19). However, this study extends prior research in two ways. First, it used DigComp 2.2 to conceptualize digital competence as a multidimensional construct rather than relying only on internet use, device ownership, or general digital literacy. Second, it used the HLQ to examine health literacy as nine independent domains rather than a single total score. This approach allowed us to identify which aspects of health literacy were more closely associated with digital competence.

Several explanations may account for the observed patterns. Information and data literacy may help older learners search for health information, compare different sources, and evaluate the reliability of online content. Problem solving may support their ability to address difficulties encountered when using digital health services, such as online appointment systems, health apps, or hospital service platforms. Communication and collaboration may be related to social support for health and interaction with healthcare providers, especially when older adults use digital communication tools to obtain advice from family members, peers, or health professionals. By contrast, digital content creation showed relatively weaker associations with HLQ domains, which is consistent with its having the lowest mean score among the five competence areas, indicating that older adult learners rarely engage in such activities. Nevertheless, the variable was significantly correlated with all HLQ scales, and several pathways may explain its relevance to health literacy: creating content reinforces appraisal of health information, sharing experiences supports social connection and active health management, and higher-order content creation may empower older adults as active health information producers. The weaker associations likely reflect low prevalence rather than conceptual irrelevance.

### 4.5 Contributions and future research

This study contributes to the literature by linking DigComp 2.2-based digital competence with multidimensional health literacy among older adult learners in China. It provides empirical evidence that digital competence is associated with several specific health literacy domains, especially those related to health information appraisal, information seeking, healthcare navigation, and understanding health information. The findings also highlight Universities of the Third Age as promising public health learning settings for addressing digital exclusion and health literacy needs among older adults.

Future research should build on these findings in several ways. First, longitudinal studies are needed to clarify the temporal relationship between digital competence and health literacy. Second, intervention studies or randomized controlled trials should evaluate whether integrated digital skills and health education programs can improve specific HLQ domains. Third, future studies should include older adults from different regions, educational backgrounds, and social participation levels to examine generalizability. Fourth, qualitative studies may help explore how older adults experience digital health barriers in real-life contexts and how programs based in Universities of the Third Age can be designed to meet their needs.

### 4.6 Limitations

This study has several limitations. First, the cross-sectional design limits causal inference. Although digital competence was positively associated with several HLQ domains, the temporal direction of these associations cannot be determined. Older adults with higher health literacy may be more motivated or better able to develop digital competence, and both digital competence and health literacy may be influenced by other factors. Future longitudinal and intervention studies are needed to clarify causal pathways and examine whether improvements in digital competence can lead to changes in specific health literacy domains.

Second, participants were recruited from four public community Universities of the Third Age in Shanghai using convenience sampling. Learners from Universities of the Third Age may be more socially active, more motivated to learn, and more educated than the general older adult population. The sample also included a relatively large proportion of younger older learners aged 55–69 years. Therefore, the findings may not be generalizable to older adults who are socially isolated, have lower educational attainment, live in rural areas, or have more severe functional or cognitive limitations. However, this sample is appropriate for examining Universities of the Third Age as educationally reachable settings for future digital health literacy interventions.

Third, all measures were based on self-reported questionnaires, which may be subject to recall bias and social desirability bias. Digital competence was assessed using a self-report DigComp 2.2-based scale rather than objective performance tasks. In addition, some potentially relevant factors, such as prior digital training, frequency of smartphone use, family support, cognitive function, and prior experience with digital health services, were not fully assessed. Future studies could combine self-reported measures with performance-based assessments, qualitative interviews, and more detailed measures of digital health service use.

## 5 Conclusion

This cross-sectional study examined the association between DigComp 2.2-based digital competence and multidimensional health literacy among older adult learners in Shanghai Universities of the Third Age. By using the five competence areas of DigComp 2.2 and the nine independent domains of the HLQ, this study provides a domain-specific understanding of how digital competence is related to health literacy in later-life learning settings.

The findings showed that digital competence was positively associated with several health literacy domains, particularly appraisal of health information, ability to find good health information, navigating the health care system, and understanding health information well enough to know what to do. These results suggest that digital competence may be especially relevant to older adults' capacity to search for, evaluate, interpret, and use health information in increasingly digitalized health environments.

From a public health perspective, the findings highlight Universities of the Third Age as potential community-based learning platforms for supporting digital inclusion and health literacy among older adults. Integrating digital skills training with health education in Universities of the Third Age may help address barriers to health information access, strengthen older learners' ability to use digital health resources, and contribute to healthy aging. However, because of the cross-sectional design, causal relationships cannot be inferred. Future longitudinal and intervention studies are needed to determine whether improving digital competence can lead to sustained improvements in specific health literacy domains and related health outcomes.

## Data Availability

The raw data supporting the conclusions of this article will be made available by the authors, without undue reservation.
